
JOURNAL INFORMATION
==============================
NLM Title Abbreviation: ScientificWorldJournal
Journal ID: ScientificWorldJournal
NLM Journal ID: 101131163
Journal ID: TSWJ

The Scientific World Journal

ISSN: 2356-6140
EISSN: 1537-744X
Publisher: Wiley

ARTICLE INFORMATION
==============================
PMCID: PMC5917145
PMID: 16937009
DOI: 10.1100/tsw.2006.201
Article ID: 530408
Article version: 1
Subjects: Directions in Science

Regulated Shedding of Syndecan Ectodomains by Chemokines

Charnaux Nathalie 1 2
Sutton Angela 1
Brule Severine 1
Gattegno Liliane 1 2 *
1 EA 3410, UFR SMBH Université Paris 13, 74 rue Marcel Cachin, 93017 Bobigny Cedex, France
2 Laboratoire de Biochimie, Hôpital Jean Verdier, AP-HP, 93143 Bondy Cedex, France
*Liliane Gattegno: liliane.gattegno@jvr.aphp.frAcademic Editor: Martin Goette

Publication date: 2006
Electronic publication date: 2006 Aug 25
Volume: 6
First page: 1037
Last page: 1040
Received 2006 Jul 5; Revised 2006 Aug 7; Accepted 2006 Aug 14
Copyright: Copyright © 2006 Nathalie Charnaux et al.
Copyright year: 2006
License: This is an open access article distributed under the Creative Commons Attribution License, which permits unrestricted use, distribution, and reproduction in any medium, provided the original work is properly cited.
License URL: https://creativecommons.org/licenses/by/3.0/

Keywords: chemokine, SDF-1/CXCL12, syndecan, heparan sulfate proteoglycan, shedding

Funding: Université Paris XIII

==============================
Directions in Science
*TheScientificWorldJOURNAL* (2006) 6, 1037–1040
ISSN 1537-744X; DOI 10.1100/tsw.2006.201

### Regulated Shedding of Syndecan Ectodomains by Chemokines

Nathalie Charnaux[1,2], Angela Sutton[1], Severine Brule[1], and Liliane Gattegno[1,2,]*
*[1]EA 3410, UFR SMBH – Université Paris 13, 74 rue Marcel Cachin, 93017 Bobigny Cedex, France; [2]Laboratoire de Biochimie, Hôpital Jean Verdier, AP-HP, 93143 Bondy Cedex, France*

**E-mail**: liliane.gattegno@jvr.aphp.fr

*Received July 5, 2006; Revised August 7, 2006; Accepted August 14, 2006; Published August 25, 2006*

---

**KEYWORDS**: chemokine, SDF-1/CXCL12, syndecan, heparan sulfate proteoglycan, shedding

---

Chemokines are chemotactic cytokines that govern multiple aspects of host defense[1]. Interest in chemokines has increased as a result of their emerging role in immune and inflammatory responses, hematopoeisis, HIV infection[2,3,4], cancer cell proliferation, and migration[5]. Chemokines mediate biological activities through activation of G-protein-coupled receptors (GPCRs)[6]; however, they also bind to glycosaminoglycans (GAGs)[7,8,9], especially heparan sulfate (HS)[10]. GAGs are long, linear, and heterogeneous sulfated polysaccharides that are generally closely associated with or immobilized on cell surfaces by attachment to protein cores, forming proteoglycans (PGs)[11]. Four chemokine subgroups have been named C, CC, CXC, or $CX_3C$ according to the number and spacing of cysteine residues[1]. Stromal cell-derived factor-1 (SDF-1/CXCL12), a CXC chemokine, constitutively expressed in a wide variety of tissues, binds to the GPCR CXCR4[6,12,13]. CXCR4 also functions as a coreceptor for X4 HIV isolates[14] and SDF-1/CXCL12 blocks cellular entry of these isolates[4,14]. Optimal inhibition requires SDF-1 interaction with cell surface HS[15]. However, the SDF-1/CXCL12-CXCR4 axis is involved in other pathological processes: angiogenesis, invasiveness, migration, and proliferation of cancer cells[16,17]. Nevertheless, the pathophysiological roles of chemokine-GAG interactions have not yet been elucidated.

In one of our recent works published in *Glycobiology*, we characterized the cell PG that binds to SDF-1/CXCL12[18]. The syndecans are a PG family, which together with the lipid-linked glypicans, are the major source of cell surface HS[11]. By way of their HS chains, syndecans bind a wide variety of ligands[11]. These PGs were also identified as HIV attachment receptors[19]. In this context, we showed that SDF-1/CXCL12 forms complexes on the human epidermoid carcinoma HeLa cell line, and on human primary lymphocytes and monocyte-derived macrophages (MDM), which comprise CXCR4 as expected and syndecan-4 (SDC-4), but not other PGs, syndecan-1 (SDC-1), CD44, nor betaglycan[18]. We also demonstrated that while SDC-4 and CXCR4 form a heteromeric complex on these cells, SDF-1/CXCL12 directly binds SDC-4 in a GAG-dependent manner. This suggests that SDF-1/CXCL12 may specifically bind a GAG structure present on SDC-4. However, this does not exclude that SDF-1/CXCL12 may also interact with some domains of the protein core of SDC-4. Moreover, in another recent paper published in *FEBS Journal*, we showed that SDC-4 behaves as a specific SDF-1/CXCL12 receptor, involved in SDF-1/CXCL12–induced transduction pathways. By specifically reducing SDC-4 expression using RNA

*Corresponding author.
©2006 with author.
Published by TheScientificWorld, Ltd.; www.thescientificworld.com interference, we demonstrated that Erk1/2 and JNK/SAPK MAPK activation by SDF-1/CXCL12 requires SDC-4 expression. Moreover, SDF-1/CXCL12 induces the phosphorylation of its GPCR, CXCR4, and also that of SDC-4[20].

Shed syndecan ectodomains are newly defined mediators of inflammation that may be involved in cell proliferation and in several regulatory processes[21,22]. It was shown that matrix metalloproteinases (MMPs) are involved in syndecan shedding and catabolic processes of syndecans[23,24]. MMPs are secreted as latent enzymes and require proteolytic cleavage for activation. Chemokine stimulation of cell MMPs have been described. For instance, SDF-1/CXCL12 stimulates the production of MMP-9 by murine RAW cells[25]. We have consequently investigated whether SDF-1/CXCL12 accelerates the shedding of PG ectodomains from human cell lines and primary cells, and tried to elucidate which transduction pathways and protease(s) are involved and whether SDF-1/CXCL12 forms complexes with the shed ectodomains of PGs.

In a recent study published in *Glycobiology*[26], we demonstrated that SDF-1/CXCL12 accelerates the shedding of SDC-4 and, to a lesser extent, that of SDC-1 from HeLa cells and MDM (Fig. 1). Syndecan shedding accelerated by SDF-1/CXCL12 depends on cell surface HS chains, since it was abolished in heparitinase-treated cells, but does not depend on CXCR4 expression, as CXCR4 silencing by RNA interference had no effect. However, this SDF-1/CXCL12–induced shedding depends on a PKC transduction pathway because it was inhibited by a PKC inhibitor. In this context, we showed that SDF-1/CXCL12 increases MMP-9 mRNA level and MMP-9 activity in HeLa cells, and that MMP-9 silencing by RNA interference strongly decreases SDC-1 and SDC–4 ectodomain shedding accelerated by SDF-1/CXCL12. Moreover, the shedding of SDC-1 and SDC–4 accelerated by SDF-1/CXCL12 from MDM was significantly inhibited by anti-MMP-9 antibodies. Therefore, MMP-9 is involved in the accelerated shedding of syndecans induced by SDF-1 in both a tumoral cell line and in human primary macrophages. However, the fact that the accelerated shedding of SDC-1 was not decreased in MMP-9–deficient mice during allergic lung infection[27] suggests that different shedding sites may be used in this PG according to the chemokine, in a tissue-dependent manner, and also possibly in a disease-specific manner. Strikingly, immunoprecipitation experiments showed us that while SDF-1/CXCL12 binds to membrane-anchored SDC-4, it does not bind to SDC-1 or SDC-4 ectodomains shed from HeLa cells. Therefore, membrane association of SDC-4 may well be critical for its interaction with SDF-1/CXCL12. In addition, the high density of SDC-4 and cell surface association may induce particular GAG chain orientation leading to optimal SDF-1/CXCL12 binding.

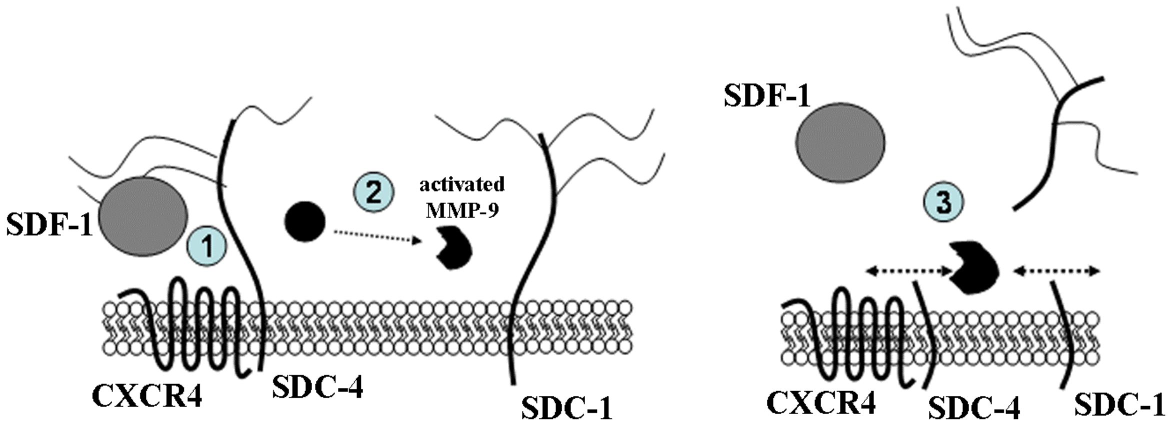

**FIGURE 1.** The shedding of SDC-4 and SDC-1 from HeLa cells and human primary macrophages is accelerated by SDF-1/CXCL12 and mediated by MMP-9. (1) SDF-1 binding to SDC-4 facilitates its presentation to CXCR4; (2) SDF-1 activates MMP-9, which is involved in syndecan shedding; (3) decreased membrane expression of syndecans down-regulates SDF-1 binding to the cells.

In summary, our data strongly indicate that the shedding of SDC-4 and SDC-1 accelerated by SDF-1/CXCL12 from human epidermoid carcinoma cells and from human primary macrophages is mediated by MMP-9. Considering that in our experiments, syndecan shedding accelerated by SDF-1/CXCL12 is associated with decreased syndecan membrane expression, as assessed by cytofluorimetric analysis after specific labeling, and that SDF-1/CXCL12 does not bind to soluble syndecan ectodomain, the role of syndecan shedding in the biological effect of SDF-1 may then be related to changes in the target cell surface phenotype rather than to a competitive inhibition in the binding of the chemokine to the target cell surface. In fact, one can speculate that SDC-4 shedding induced by SDF-1 /CXCL12 and mediated by MMP-9 could be part of an autoregulatory/down-regulation cycle: (1) SDF-1/CXCL12 binding to SDC-4 facilitates its presentation to CXCR4; (2) SDC-4 is a signaling molecule for SDF-1/CXCL12; (3) while SDF-1 accelerates SDC-4 ectodomain shedding, this chemokine decreases SDC-4 membrane expression. Therefore, SDF-1/CXCL12 down-regulates the cell plasma membrane expression of its coreceptor, the SDC-4. Interestingly, we recently demonstrated that RANTES/CCL5 (1) accelerates the shedding of SDC-1 and SDC-4 from HeLa cells, which depends on CCR5 and on both Erk1/2 MAPK and PKC transduction pathways; and (2) forms GAG-dependent complexes with the shed ectodomains of these PGs[28]. In addition, Xu et al. showed that SDC-1 ectodomains bind to CCL-7, -11, and -17 during lung inflammation[27]. Therefore, the respective molecular events involved in the acceleration of shedding of PGs induced by RANTES/CCL5 or SDF-1/CXCL12 differ according to the chemokine. Moreover, the binding capability of chemokines to shed syndecan ectodomains differs. For instance, RANTES/CCL5 and SDF-1/CXCL12, respectively, CC- and CXC-chemokines, are quite different. They show different GAG-binding epitopes and different quaternary structure (dimeric and oligomeric states)[29]. They also exhibit different expression patterns. RANTES is preferentially secreted during inflammatory process, whereas SDF-1/CXCL12 is constitutively expressed in various cell types and may play a “homeostatic” role. Therefore, SDC-1 and SDC-4 shedding accelerated by RANTES/CCL5 could be expected to occur during inflammatory and wound repair processes, whereas PG shedding accelerated by SDF-1/CXCL12 could occur even in the absence of any tissue injury. This accelerated shedding by chemokines may, therefore, represent fine regulatory mechanisms of chemokine activity in physiology and diseases.

### ACKNOWLEDGMENTS

This work was supported by the Direction de la Recherche et des Enseignements Doctoraux (Ministère de l’Enseignement Supérieur et de la Recherche), Université Paris XIII.

### REFERENCES

1. Luster, A.D. (1998) Chemokines--chemotactic cytokines that mediate inflammation. *N. Engl. J. Med.* **338**, 436–445.
2. Baggiolini, M., Dewald, B., and Moser, B. (1997) Human chemokines: an update. *Annu. Rev. Immunol.* **15**, 675–705.
3. Ward, S.G., Bacon, K., and Westwick, J. (1998) Chemokines and T lymphocytes: more than an attraction. *Immunity* **9**, 1–11.
4. Bleul, C.C., Wu, L., Hoxie, J.A., Springer, T.A., and Mackay, C.R. (1997) The HIV coreceptors CXCR4 and CCR5 are differentially expressed and regulated on human T lymphocytes. *Proc. Natl. Acad. Sci. U. S. A.* **94**, 1925–1930.
5. Strieter, R.M. (2001) Chemokines: not just leukocyte chemoattractants in the promotion of cancer. *Nat. Immunol.* **2**, 285–286.
6. Schwarz, M.S. and Wells, T.N. (1999) Interfering with chemokine networks—the hope for new therapeutics. *Curr. Opin. Chem. Biol.* **3**, 407–417.
7. Hoogewerf, A.J., Kuschert, G.S., Proudfoot, A.E., Borlat, F., Clark-Lewis, I., Power, C.A., and Wells, T.N. (1997) Glycosaminoglycans mediate cell surface oligomerization of chemokines. *Biochemistry* **36**, 13570–13578.
8. Lortat-Jacob, H., Grosdidier, A., and Imberty, A. (2002) Structural diversity of heparan sulfate binding domains in chemokines. *Proc. Natl. Acad. Sci. U. S. A.* **99**, 1229–1234.
9. Martin, L., Blanpain, C., Garnier, P., Wittamer, V., Parmentier, M., and Vita, C. (2001) Structural and functional analysis of the RANTES-glycosaminoglycans interactions. *Biochemistry* **40**, 6303–6318.
10. Sadir, R., Baleux, F., Grosdidier, A., Imberty, A., and Lortat-Jacob, H. (2001) Characterization of the stromal cellderived factor-1alpha-heparin complex *J. Biol. Chem.* **276**, 8288–8296.
11. Bernfield, M., Gotte, M., Park, P.W., Reizes, O., Fitzgerald, M.L., Lincecum, J., and Zako, M. (1999) Functions of cell surface heparan sulfate proteoglycans. *Annu. Rev. Biochem.* **68**, 729–777.
12. Coulomb-L'Hermin, A., Amara, A., Schiff, C., Durand-Gasselin, I., Foussat, A., Delaunay, T., Chaouat, G., Capron, F., Ledee, N., Galanaud, P., Arenzana-Seisdedos, F., and Emilie, D. (1999) Stromal cell-derived factor-1 (SDF-1) and antenatal human B cell lymphopoiesis: expression of SDF-1 by mesothelial cells and biliary ductal plate epithelial cells. *Proc. Natl. Acad. Sci. U. S. A.* **96**, 8585–8590.
13. Pablos, J.L., Amara, A., Bouloc, A., Santiago, B., Caruz, A., Galindo, M., Delaunay, T., Virelizier, J.L., and Arenzana-Seisdedos, F. (1999) Stromal-cell derived factor is expressed by dendritic cells and endothelium in human skin. *Am. J. Pathol.* **155**, 1577–1586.
14. Oberlin, E., Amara, A., Bachelerie, F., Bessia, C., Virelizier, J.L., Arenzana-Seisdedos, F., Schwartz, O., Heard, J.M., Clark-Lewis, I., Legler, D.F., Loetscher, M., Baggiolini, M., and Moser, B. (1996) The CXC chemokine SDF-1 is the ligand for LESTR/fusin and prevents infection by T-cell-line-adapted HIV-1. *Nature* **382**, 833–835.
15. Valenzuela-Fernandez, A., Palanche, T., Amara, A., Magerus, A., Altmeyer, R., Delaunay, T., Virelizier, J.L., Baleux, F., Galzi, J.L., and Arenzana-Seisdedos, F. (2001) Optimal inhibition of X4 HIV isolates by the CXC chemokine stromal cell-derived factor-1alpha requires interaction with cell surface heparan sulfate proteoglycans. *J. Biol. Chem.* **276**, 26550–26558.
16. Geminder, H., Sagi-Assif, O., Goldberg, L., Meshel, T., Rechavi, G., Witz, I.P., and Ben-Baruch, A. (2001) A possible role for CXCR4 and its ligand, the CXC chemokine stromal cell-derived factor-1, in the development of bone marrow metastases in neuroblastoma. *J. Immunol*. **167**, 4747–4757.
17. Kryczek, I., Lange, A., Mottram, P., Alvarez, X., Cheng, P., Hogan, M., Moons, L., Wei, S., Zou, L., Machelon, V., Emilie, D., Terrassa, M., Lackner, A., Curiel, T.J., Carmeliet, P., and Zou, W. (2005) CXCL12 and vascular endothelial growth factor synergistically induce neoangiogenesis in human ovarian cancers. *Cancer Res*. **65**, 465–472.
18. Hamon, M., Mbemba, E., Charnaux, N., Slimani, H., Brule, S., Saffar, L., Vassy, R., Prost, C., Lievre, N., Starzec, A., and Gattegno, L. (2004) A syndecan-4/CXCR4 complex expressed on human primary lymphocytes and macrophages and HeLa cell line binds the CXC chemokine stromal cell-derived factor-1 (SDF-1). *Glycobiology* **14**, 311–323.
19. Saphire, A.C., Bobardt, M.D., Zhang, Z., David, G., and Gallay, P.A. (2001) Syndecans serve as attachment receptors for human immunodeficiency virus type 1 on macrophages. *J. Virol.* **75**, 9187–9200.
20. Charnaux, N., Brule, S., Hamon, M., Chaigneau, T., Saffar, L., Prost, C., Lievre, N., and Gattegno, L. (2005) Syndecan-4 is a signalling molecule for stromal derived-cell factor-1 (SDF-1)/CXCL12. *FEBS J.* **272**, 1937–1951.
21. Fitzgerald, M.L., Wang, Z., Park, P.W., Murphy, G., and Bernfield, M. (2000) Shedding of syndecan-1 and -4 ectodomains is regulated by multiple signaling pathways and mediated by a TIMP-3-sensitive metalloproteinase. *J. Cell Biol.* **148**, 811–824.
22. Gotte, M. (2003) Syndecans in inflammation. *FASEB J.* **17**, 575–591.
23. Li, Q., Park, P.W., Wilson, C.L., and Parks, W.C. (2002) Matrilysin shedding of syndecan-1 regulates chemokine mobilization and transepithelial efflux of neutrophils in acute lung injury. *Cell* **111**, 635–646.
24. Endo, K., Takino, T., Miyamori, H., Kinsen, H., Yoshizaki, T., Furukawa, M., and Sato, H. (2003) Cleavage of syndecan-1 by membrane type matrix metalloproteinase-1 stimulates cell migration. *J. Biol. Chem.* **278**, 40764–40770.
25. Yu, X., Huang, Y., Collin-Osdoby, P., and Osdoby, P. (2003) Stromal cell-derived factor-1 (SDF-1) recruits osteoclast precursors by inducing chemotaxis, matrix metalloproteinase-9 (MMP-9) activity, and collagen transmigration. *J. Bone Miner. Res.* **18**, 1404–1418.
26. Brule, S., Charnaux, N., Sutton, A., Ledoux, D., Chaigneau, T., Saffar, L., and Gattegno, L. (2006) The shedding of syndecan-4 and syndecan-1 from HeLa cells and human primary macrophages is accelerated by SDF-1/CXCL12 and mediated by the matrix metalloproteinase-9. *Glycobiology* **16**, 488–501.
27. Xu, J., Park, P.W., Kheradmand, F., and Corry, D.B. (2005) Endogenous attenuation of allergic lung inflammation by syndecan-1. *J. Immunol.* **174**, 5758–5765.
28. Charnaux, N., Brule, S., Chaigneau, T., Saffar, L., Sutton, A., Hamon, M., Prost, C., Lievre, N., Vita, C., and Gattegno, L. (2005) RANTES (CCL5) induces a CCR5-dependent accelerated shedding of syndecan-1 (CD138) and syndecan-4 from HeLa cells and forms complexes with the shed ectodomains of these proteoglycans as well as with those of CD44. *Glycobiology* **15**, 119–130.
29. Handel, T.M., Johnson, Z., Crown, S.E., Lau, E.K., Sweeney, M., and Proudfoot, A.E. (2005) Regulation of protein function by glycosaminoglycans - as exemplified by chemokines. *Annu. Rev. Biochem.* **74**, 385–410.

**This article should be cited as follows:**

Charnaux, N., Sutton, A., Brule, S., and Gattegno, L. (2006) Regulated shedding of syndecan ectodomains by chemokines. *TheScientificWorldJOURNAL* **6**, 1037–1040. DOI 10.1100/tsw.2006.201.
